# Understanding User Involvement in Research in Aging and Health

**DOI:** 10.1177/2333721419897781

**Published:** 2019-12-29

**Authors:** Susanne Iwarsson, Anna-Karin Edberg, Synneve Dahlin Ivanoff, Elizabeth Hanson, Håkan Jönson, Steven Schmidt

**Affiliations:** 1 Lund University, Sweden; 2 Kristianstad University, Sweden; 3 Gothenburg University, Sweden; 4 Linnaeus University, Kalmar, Sweden; 5 Swedish Family Care Competence Centre, Kalmar, Sweden

**Keywords:** community, public and patient involvement, disability, gerontology, nursing, public health/public policy, rehabilitation, social work, volunteering, frail older adults

## Abstract

User involvement in research is advocated as an avenue for efficient societal developments. In this article, we identify potentials, problems, and challenges related to research on aging and health, and identify and illustrate research priorities using an evolving research program as an example. Involving user representatives in the development phase, the UserAge program engages researchers at four universities in Sweden. The program builds upon previous and ongoing research with user involvement. The goals are to maximize the impact of user involvement, enhance the execution of high-quality research, increase the knowledge about what difference user involvement can make, and evaluate the impact of research about and with user involvement. Taken together and communicated in the international scientific community as well as in a wide range of public arenas, the empirical results, capacity-building, and modeling efforts of UserAge will have an impact not only on the present situation but also on the future.

## Introduction

Addressing active and healthy aging and the challenges in welfare systems demands new solutions. While top-down centrally driven solutions dominate in many countries, policy makers emphasize the need for bottom-up approaches in which users have to be involved (Walker, 2007). User involvement is being advocated, but in comparison with fields such as disability and mental health, aging and health research is lagging behind. The needs and preferences of the diverse aging population are new, rapidly changing, and far from being met by society. Older adults want greater choice and the possibility to influence the services and products they need. At the same time, there are vulnerable groups whose voices are seldom heard (Backhouse et al., 2016; Österholm, 2016).

The rationale for involving users in research has its origin in ideas of empowerment, with a striving to shift power in the research process from researchers to users (Mockford et al., 2012). The research process should be carried out with genuine user involvement to reveal dilemmas and controversial issues and deal with them constructively. Research has seen a strong progression, but the translation of new knowledge to practice is slow. The impact in terms of effects on, changes for, or benefits to society remains to be demonstrated. With this article, we aspire to identify potentials, problems, and challenges related to user involvement in research on aging and health, and to identify and illustrate research priorities using an evolving research program as an example.

### Concepts and Theory

User-oriented research showcases a plethora of conceptual and theoretical foundations, and the fundamental approaches relating to the theory of the science require clarification (Kylberg et al., 2018). A variety of terms are used, such as user involvement, user-driven research, community-based participatory research, co-design, participatory design, co-production of knowledge, patient and public involvement, patient-driven research, transdisciplinary research, and collaborative research. Against this background, it is difficult to gain a comprehensive overview of the state-of-the-art regarding user involvement in research on aging and health. Moreover, there is a paucity of theoretical support in the literature.

Problematized already by Arnstein (1969), user involvement can be described in stages ranging from nonparticipation, through tokenism, and, finally, to citizen control. In its widest sense, concepts and models on user involvement could be related to a possible shift toward context-driven problem-focused research that Gibbons et al. (1994) referred to as “Mode 2 knowledge-production,” and to a debate concerning governance and integrity of research (Fuller, 2000). User involvement may be about involving users in research processes (Gradinger et al., 2013; Shippee et al., 2013; Ståhl et al., 2008) or, alternatively, in the development of health care (Ross et al., 2014).

### Who Are the Users?

In research applying public involvement, the user is an active partner involved in key discussions and decisions, sharing his or her unique knowledge, expertise, and perspective (Staniszewska et al., 2018) rather than being a study object, but there are fundamental differences in the basic tenets and indeed in the meaning of the term “user.” Different categories of users represent an interesting but potentially problematic mix of perspectives, hitherto seldom spelled out or specifically addressed in practice or in research. One category is “service users,” which is applicable in quality development and research concerning health care and social services. The World Health Organization (WHO, 2012) has recommended a broad definition of “knowledge users” including all parties that are interested in and/or beneficiaries of the new knowledge produced from research on aging and health. Examples are senior citizens in general as well as vulnerable people with specific characteristics and needs; informal carers; health care, social services, physical planning, and industry professionals; and public agency, policy maker, and interest organization representatives. Applying a critical stance to the term knowledge users, there might be a risk that it communicates an idealized image of a harmonious production and use of scientific knowledge. Examples of additional questions are how the term relates to “stakeholders” and are not researchers themselves the primary users of knowledge? Pointing to yet another term, older people are often targeted as “product users” or “end users” when studying new technologies. No matter the category of users, attention to diversity in terms of gender, age, ethnicity, and socioeconomic factors as well as physical, psychological, and intellectual functional abilities is warranted. Overall, the attention to the definition of different categories of users is insufficient, and there is a need for research fostering the development of a more coherent terminology based on clear definitions.

### Pros and Cons of User Involvement

Several authors recommend user involvement at all stages of the research process (Joss et al., 2015), while others emphasized that such research operates along a continuum with pros and cons at each level of involvement (Lowes & Hulatt, 2005). Participatory approaches raise a number of issues among users (Dent & Pahor, 2015), and there are also challenges and tensions in issues of integrity, equality, equivalence, legal certainty, transparency, efficiency, and distribution of authority (Joss et al., 2015; Tritter & McCallum, 2006). One challenge lies in conducting the dialogue on equal terms and converting users’ expressed problem areas or ideas into issues that can be scientifically studied (Erdtman et al., 2012). A further dilemma concerns the recruitment of user representatives. Who should be asked, who assumes the role of user, and whom can they represent? Additional challenges have been identified with involving users in data collection (Priestley et al., 2010) and analysis (Cotterell, 2008).

Turning to the positive effects and outcomes of user involvement, Brett et al. (2014) identified three types of effects: for the individual user, for the category of citizens affected, and for the researchers. Reported outcomes are better methodological and ethical quality, increased relevance and impact, effective dissemination of findings, and improved health outcomes (Gradinger et al., 2013). However, the effects are complex and complicated to evaluate (Barber et al., 2011), and there remains a dearth of studies about user involvement in research (Kylberg et al., 2018).

### Research Priorities

Overall, previous and current aging and health research involving users makes a split impression. A major reason is that the majority of publications in this field report research with user involvement, while few publications focus on research about user involvement. Occasionally, both perspectives are represented within one and the same publication without explicitly spelling out or differentiating between them (Kylberg et al., 2018). As previously mentioned, there is insufficient attention given to the definition of different categories of users involved in reported studies. Conceptual definitions and theory development, alongside methodological development using a wide range of approaches and study designs, are needed. Most existing research relies heavily on qualitative designs, while quantitative studies producing generalizable results are scarce. Specifically, there is a need to develop valid methods to evaluate research with user involvement. Furthermore, user involvement in research has introduced new ethical challenges and dilemmas that have not been sufficiently addressed, and the resources needed for research with user involvement deserve consideration. As users are seldom actively involved in knowledge translation (KT) activities (Joss et al., 2015) but rather the recipients of information presented by researchers, research focusing on such aspects is also called for.

### Development of a Research Program With and About User Involvement

Using these priorities as a starting point, we developed the research program used for exemplification in this article. Making use of already established networks, we set an interactive process into action involving researchers from four universities in South Sweden and user representatives. Starting 6 months prior to the deadline for the targeted call, we formed a constellation of experienced researchers with leadership and grant management skills and experience, with one assigned the status as principal investigator (PI). Administrative capacity to support the proposal process was included. Next, we committed a team of senior and junior researchers representing a range of different disciplines. A heterogeneous group of knowledge users was formed and instructed to be prepared to give input during the proposal preparation phase. Three international scientific experts were engaged to serve as critical friends during the proposal preparation process.

**Figure 1.**
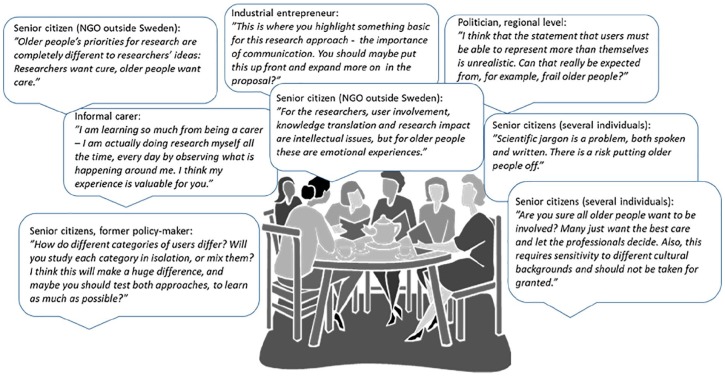
Examples of user input to the UserAge Program proposal.

The iterative process included personal and online meetings in different constellations involving the user representatives individually or as a group. A 2-day retreat engaging all researchers and user representatives, as well as international consultants, was the most intensive and productive element. During this meeting, we discussed research questions, methodology, and program structure, which resulted in a complex draft proposal. Users communicated during and after the retreat that it was interesting but highly demanding, and they felt that it was difficult for them to give the kind of input they wanted in this context. As we aspired to ensure further input to the evolving proposal, the PI provided several user representatives with a popularized summary of the proposal and interviewed them individually, adapting the situation and requests for input to each person engaged. For examples of user input to the proposal at this stage, see Figure 1. Eventually, the proposal was submitted and later awarded funding (SEK 18 million for years 2017–2022).

### The UserAge Program

**Figure 2.**
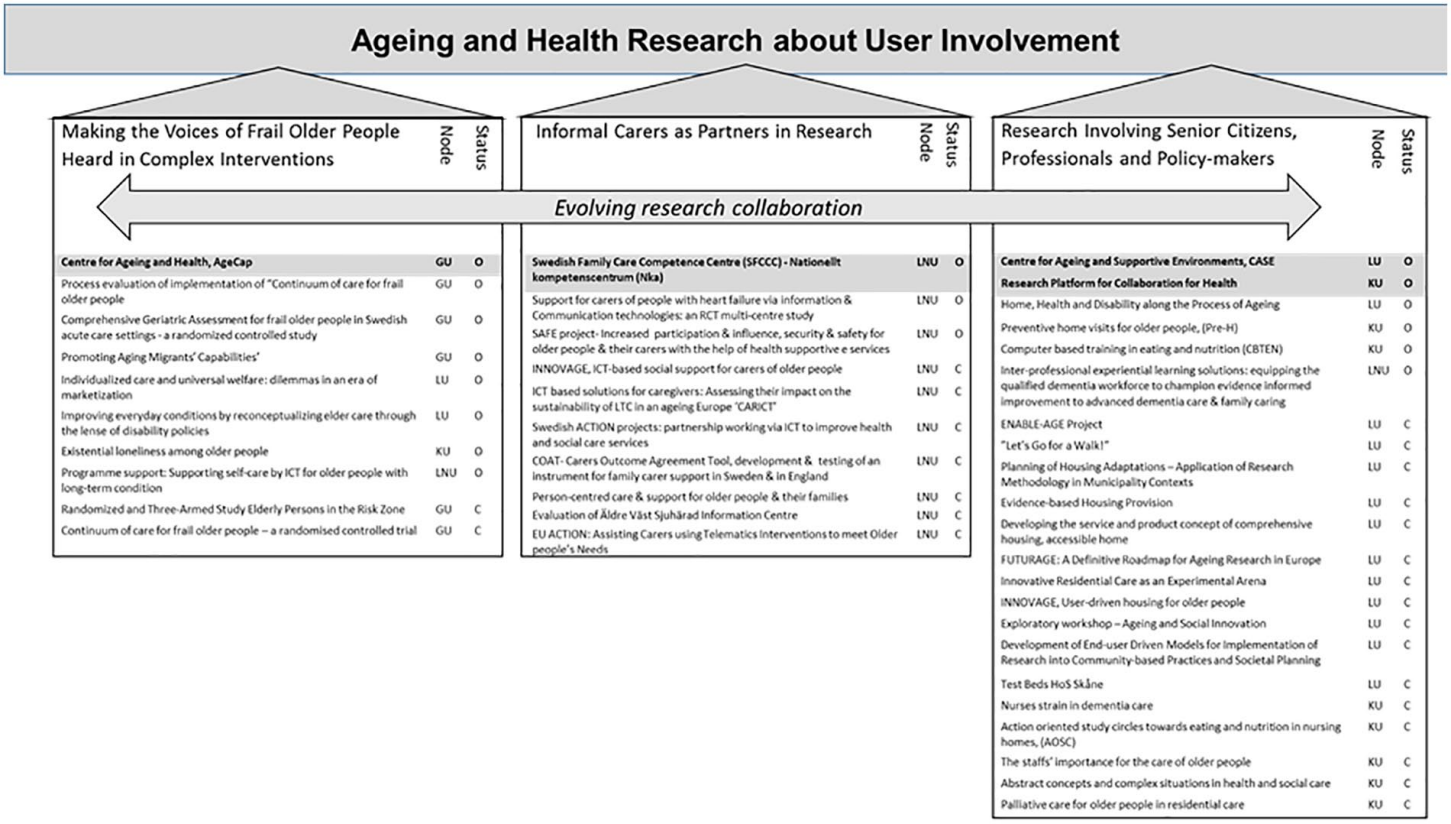
Overview of research with user involvement underlying the UserAge Program proposal. *Note.* GU = University of Gothenburg; LU = Lund University; KU = Kristianstad University; LNU = Linnaeus University; O = ongoing; C = completed.

The program builds upon the consortium’s previous and ongoing research with user involvement (Figure 2) and focuses on the involvement of different categories of knowledge users in research on aging and health. Carefully prepared in collaboration between the researchers and user representatives, the goals are to maximize the impact of user involvement, enhance the execution of high-quality research, increase the knowledge about what differences user involvement can make, and evaluate the impact of research about and with user involvement. On an empirical level, the aims are to enhance the execution of high-quality research and increase the knowledge about what differences user participation in the research process can make. On the capacity-building and modeling levels, the aims are to develop methodology, training, and a model for KT as well as a theoretical model directing the evolution of user participation in research on aging and health. Examples of research questions guiding our work are presented in Table 1.

**Table 1.** Examples of UserAge Research Questions.

UserAge Program component	Research question
PhD Student Projects	What are the significant meaningfulness, challenges, and opportunities of user involvement for frail older people, informal carers, senior citizens, professionals, and policy makers—at individual, group, and societal levels?
Panel Study	What are the awareness of, understanding of, and attitudes to user involvement among different categories of users and researchers in Sweden? What are the changes over time?
Cross-Cutting Studies	What are the needs for and relevance of user involvement in research? What are the dilemmas and controversies related to the tension between researchers with their striving for integrity and independent scientific thinking and the empowerment and feelings of being valued as users and actors in research? How can such tension be attended to, resolved, and used to the benefit of knowledge creation and KT? What are the ethical and legal dilemmas and controversies of user involvement in research, and how could they be satisfactorily dealt with? How does user involvement affect (a) different categories of users and researchers, and (b) the outcomes and impact of research? What is the meaningfulness, validity, trustworthiness, and effectiveness of different methods for participation in research for different categories of users?
KT and Research Impact	What are the opportunities and challenges inherent in different communication channels and modes for KT involving different categories of users and researchers?

*Note.* KT = knowledge translation.

**Figure 3.**
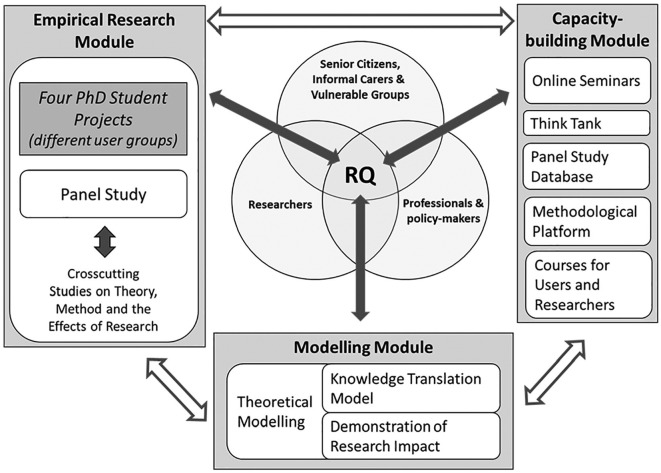
Overview of the UserAge Program. *Note.* RQ = research questions.

Starting out from the jointly identified research priorities and research questions, we established an empirical research module, a capacity-building module, and a modeling module that build up the UserAge program (Figure 3). Integrated in the management, a User Board and an External Advisory Committee composed of researchers and users give important input and monitor the overall development of the program.

### Empirical Research Module

Four PhD student projects outlined in Table 2 are at the core of this module (see Figure 3), and they address specific categories of knowledge users: frail older people; informal carers; staff in health care, social services, and public multifamily housing; policy makers; and the general population of senior citizens. In addition to focusing on specific categories of users, one of these projects includes cross-cutting facets addressing user involvement as a phenomenon. The majority of these ongoing or planned studies are based on qualitative or mixed-methods designs.

**Table 2.** Overview of the Four PhD Student Projects in the UserAge Program.

Project title	Sub-study	Study design
Making the Voices of Frail Older People Heard in Complex Interventions	1. Exploring the meaning of research to frail older people	Qualitative
	2. Health care personnel’s perspective on user involvement in research for frail older people	Qualitative
	3. Researchers’ perspective on user involvement in intervention research for frail older people	Qualitative
	4. Enabling frail older people to make their voices heard	Qualitative
Informal Carers as Partners in Research	1. Moving beyond the first response phenomenon—exploring carers’ views and experiences of being involved in research and development work	Qualitative
	2. How carer characteristics influence their views, awareness, and understanding of involvement in research	Quantitative
	3. The significance of carers as partners in co-designing a support intervention	Mixed methods
	4. Practitioners, decision makers, and policy makers’ views and experiences of involving carers in research on aging and health	Delphi study
Health Care, Social Services Staff, and Policy Makers in Collaboration With Researchers	1. Exploring health professionals’ experiences of being involved in a research project—a case study	Qualitative
	2. Conceptualizing researchers’ perspectives on what involvement of professionals in research can lead to—a Group Concept Mapping study	Mixed methods
	3. Conceptualizing professionals’ perspectives on what involvement of professionals in research can lead to—a Group Concept Mapping study	Mixed methods
	4. Developing an instrument to measure user involvement	Quantitative
Understanding the Phenomenon of User Involvement Through Senior Citizens and Staff in Public Multifamily Housing	1. Critical aspects in decision making related to housing accessibility and aging	Qualitative
	2. The development of user involvement in the context of an interdisciplinary research center	Qualitative
	3. Senior citizens’ experiences and attitudes to user involvement in research	Quantitative
	4. User involvement in a mass-experiment targeting housing accessibility for people aging with disability	Mixed methods

Adding a major quantitative component, the empirical module includes a national Panel Study targeting different categories of knowledge users as well as researchers. Launched in August 2019, the aim of this longitudinal study is to capture trends in attitudes, awareness, and knowledge about user involvement in research. The panel will also serve as a recruitment base for user groups to discuss and test research ideas, methodologies, evolving results, and practical solutions within UserAge. The national random sample of 1,200 older adults in different phases of the aging process was recruited using stratified sampling techniques to ensure representation. A sample of 100 people per group was recruited from existing networks and advertisements to represent two other categories of knowledge users (informal carers and professionals in relevant sectors) and researchers in aging and health. Commissioned to a company regularly implementing national surveys, for the recruitment, we used the national population register, the member list of a national carers’ organization, health care and municipality records of staff, and the Swedish Gerontological Society as well as the researcher network of a national graduate school for research on aging and health. The survey was developed using a user forum, where researchers and user representatives jointly developed the survey questionnaire for older adults, which was subsequently adapted to fit each of the three user categories.

These five projects constitute the empirical base for methodology development and production of new knowledge. The empirical module includes several cross-cutting studies (including facets of one of the PhD student projects; see above) feeding into the Modeling module (described below; see Figure 3), and thus provides input to the generation of theory for research with and about user involvement in research on aging and health.

### Capacity-Building Module

Aiming to build future capacity for research on aging and health with user involvement, this module comprises five facets (Figure 3). The first is a series of *Online seminars* open to all co-workers in UserAge, including users. Taking turns among the senior researchers to convene these sessions, there is a specific emphasis on presentations and critical discussions of the PhD students’ work. Second, a *Think Tank on participation* is an external resource with a specific focus on participation as a phenomenon, constituting an important connecting link in the societal ambition of integrating large varieties of expertise, and as a field of tensions. Members discuss experiences and findings during meetings and workshops engaging invited international guest researchers, thus increasing the cross-disciplinary input and overall validity of the emerging new knowledge. Third, utilizing the data produced by the Panel Study, we will establish a *National database for studies on attitudes toward user involvement in research*. As this database could also be used for related research, the ambition is to open it up to other aging and health researchers. Documenting and providing information about the methods used in the empirical project, the fourth facet is a *Methodological platform*. Experienced methodologists are developing consulting capacity for UserAge researchers as well as others, and material describing different methods will be presented online as well as in a book format. Finally, as co-learning by users and researchers is necessary (Shippee et al., 2013) and there is a paucity of capacity building in the form of education and training for research with and about user involvement, we will develop *University courses for users and future aging and health researchers*. The course for users will be developed with inspiration from DePoy and Gitlin (1994), to “demystify the research process” and provide participants with a foundation from which to understand research designs and their applications. While courses may address different target groups, they will have several components in common and be arranged in formats where users and junior scholars meet and interact. This will require the development of structured yet interactive and flexible modules following the pace and issues raised by participants themselves (Buffel, 2015). Both courses will be implemented with teachers engaged in UserAge and complemented with input from international experts (researchers and users).

### Modeling Module

This module comprises three facets. Initiated already upon establishment of the program, we aspire to develop a *Model for KT*. Making use of the KT framework suggested by the WHO (2012), we are making efforts to elaborate and apply a model for KT that is able to capture the peculiarities of aging and health research. In a mutual learning process involving researchers and users in parallel with the empirical projects, we applied WHO’s model to describe and deductively categorize previous and ongoing KT strategies in use at the four UserAge partner universities. Absorbing state-of-the-art theories and methodologies specifically developed for KT in the fields of science communication, media studies, implementation science, and evaluation studies, the ongoing iterative development process includes theoretical as well as practical facets. For example, one practical task was to produce a popularized Swedish version of the program proposal to enhance communication with users. Combining our previous and ongoing work with currently available evidence, we foresee that the UserAge model for KT could be scaled up and used nationally and internationally.

This work feeds into the *Demonstration of the research impact* endeavor (Figure 3), where we will effectuate assessment and evaluation of research impact across the UserAge projects. Making use of the collective efforts of the research group (including users; Shippee et al., 2013) and the results produced, the goal is to develop a structure and strategy to evaluate research impact. We will identify the most relevant and significant new knowledge produced, and the changes we are aiming for. Making use of the case study format and experiences from the Research Excellence Framework (REF) in the United Kingdom (Manville et al., 2015), we will define an overall research impact pathway objective, starting with the impact and projections we primarily are aiming for. This will result in a compilation of case studies to be published later in the program.

Taking the first concrete steps when approaching the second half of the program period, we are engaged in theory development. It is evident that user involvement is associated with a number of challenges, and our choice has been to address these challenges as part of the knowledge production and knowledge use of the program. All co-workers (including user representatives, Think Tank members) as well as researchers external to the program will be actively engaged in a process aiming to transmit knowledge associated with theory. A particular feature is the inclusion of a number of critical studies where the gains and problems of the program itself are investigated and brought into a common process of learning, which involves researchers and users. This is facilitated by the comprehensive transdisciplinary program design, where some researchers are in training as PhD students.

We will adopt a creative, iterative theory development process including writing, presenting, critiquing, and soliciting feedback on emerging ideas (Byron & Thatcher, 2016) and make use of approaches such as case studies, thought experiments, reasoning techniques, role-playing, and other creative and inductive methods. Well-defined concepts constitute one basic condition for theory development (Jaccard & Jacoby, 2010). Identifying and defining concepts central to aging and health research about and with user involvement, articulating how these concepts are related, and explaining the underlying dynamics will be the task for a Delphi study (Hsu & Sandford, 2007). The ideas serving as the starting point for the theory development endeavor will be explicitly connected to the ongoing findings generated in the empirical projects. In addition, the Panel Study will be the recruitment base for specific studies supporting the theory development ambition. Meta-ethnographic approaches (Rycroft-Malone & Burton, 2015) that assemble the findings of multiple primary qualitative studies using a systematic approach have the potential to add breadth and depth, and thereby generate comprehensive and generalizable theory (Thomas et al., 2004). Moreover, the use of grounded theory (Charmaz, 2006) in several of the empirical projects will nurture the theory development process. Thus, we will make progress toward a theoretical model of user involvement by reinterpreting the meaning across several qualitative studies. We will also be able to derive plausible hypotheses that can be tested making use of the Panel Study data.

## Discussion

The UserAge program is a comprehensive and large-scale research initiative specifically aiming to foster the understanding of user involvement in research on aging and health, which to the best of our knowledge is internationally unique. For a long time, policy makers and funding bodies have advocated user involvement in research as an opportunity for societal development, but it is also a field of tension and paradox, hitherto insufficiently studied (O’Sullivan, 2018). Our ongoing research is generating new knowledge with the potential to inform future policy developments.

From a democratic perspective, user involvement has been assigned value in its own right with positive effects such as customized welfare systems and stimulation of citizenship. However, even with the best intentions of power sharing, researchers have an advantage based on their training and position. Participatory research is a form of power and carries the risk of reproducing the inequalities it seeks to address (Buffel, 2015). Critics claim that user involvement contains more rhetoric than substance (Shippee et al., 2013) with a potential for manipulation leading to unjust exercises of power (Hickey & Mohan, 2004). There is also a risk of excluding vulnerable groups because of difficulties reaching them and communicating effectively (Gradinger et al., 2013). In the UserAge program, we are studying user involvement in interdisciplinary research targeting several different categories of users, for example, informal carers (Andréasson et al., 2019; Malm et al., 2019). We argue that studies involving well-defined target groups are necessary to elucidate differences and similarities regarding user involvement in research in different populations, thus informing future research and development.

A diversity of researchers and users assert the value of their particular knowledge and experience in almost every area in society. Increasingly, we find a compulsion to coordinate between and within professions, scientific disciplines, organizations, and nations to reduce a vertical distance and isolation by integrating a variety of expertise, including specified, situated, and local forms of knowledge (Kinsella, 2004). It should be noted that far from all researchers are proponents of user involvement in research. With the UserAge Panel Study, we aspire to deliver results elucidating the situation among researchers in aging and health in Sweden. User involvement challenges many of the values and assumptions researchers hold (Gradinger et al., 2013), but these concerns seem to go unnoticed by policy makers. Such dilemmas and controversies deserve attention, but little is known about attitudes toward user involvement among researchers. As the positive and potentially negative effects of participatory research approaches are largely unknown and the evidence in terms of outcomes is weak (Shippee et al., 2013), gathering data not only from users but also from researchers is one avenue to increase the understanding about the tensions in this field of research. It is often argued that participatory integration would help bridge the gaps between governing and being governed, between theory and practice, and between science and everyday experience, but few major research endeavors have targeted this complexity related to research on aging and health.

Practicing user involvement in our previous research and using these experiences to develop the UserAge program (see Figure 2), we have produced new knowledge and potentially higher quality output from research that is more relevant in practice than it would have been without user involvement. Well in line with similar initiatives in other national contexts (Sixsmith et al., 2017), our studies targeting senior citizens to be more active and empowered, for example, in their search for optimal housing solutions (Haak et al., 2015; Jonsson et al., 2016) delivered knowledge on how such research can contribute to positive developments. Other studies have focused on empowering older adults with different ethnic backgrounds to take control of their own health (Gustafsson et al., 2015). Health care professionals have been in focus in action-oriented study circles, an approach shown to be successful in integrating scientific evidence in naturalistic environments for improved participant outcomes in relation to nutrition (Westergren, 2012). Yet other studies have a strong focus on development of partnerships in research with informal careers, with a specific focus on the use of information and communication mediated support (Hanson et al., 2011). Taking a step back from this empirical background (see Figure 2) to study user involvement as a phenomenon in its own right, the ongoing studies in the UserAge program represent a novel approach, which is attracting interest from users, researchers, and policy makers alike. The results will help us to understand user needs and preferences, the relevance of user involvement in research, and views and experiences of KT and research utilization. With this broad and evolving knowledge base, we will develop new user-driven research ideas that will feed into the UserAge projects.

Based on progressive initiatives in national contexts, research on public involvement and user involvement has seen a noteworthy development during the latest decades in Europe, particularly in the United Kingdom (Staniszewska et al., 2018) and Ireland (O’Sullivan, 2018). Overall, the international relevance and applicability of ongoing research about user involvement is not known, but the findings and recommendations should be transferable to other countries and continents (Staniszewska et al., 2018). As an ambitious initiative in a Nordic welfare state such as Sweden, the UserAge program will deliver results and insights adding to the global knowledge base on user involvement in research on aging and health. In addition to informing future research in this field, the new knowledge generated should be useful and of interest for practitioners and decision makers in welfare services, nongovernmental organizations, and public authorities.

Methodological issues are of significant importance (Shippee et al., 2013) because research sponsors today often ask for user involvement in their projects. Hitherto, such research is dominated by qualitative approaches, as exemplified by a recent publication reporting experiences from our previous research (Löfqvist et al., 2019). While highly valuable for developing in-depth knowledge, there is a risk that the buildup of knowledge will be a slow and scattered process. Therefore, it is necessary to develop a broader arsenal of methods that lay the foundations for the development of generalizable knowledge and cumulative knowledge building. A noteworthy strength of the UserAge program is that we are using qualitative and quantitative approaches as well as mixed methods, including state-of-the art and newly developed methods. Involving user categories with specific needs and prerequisites comes with specific challenges (Backhouse et al., 2016), which hitherto have been insufficiently explored and addressed in a rigorous manner. Representing another strength of the program, we are developing and testing new methodologies that allow for the involvement of people whom are severely restricted to participate in traditional forms of research, thus contributing to the much-needed methodology development in this field. Time and cost aspects need to be elucidated more thoroughly from the point of view of both users and researchers to create favorable conditions for future research, and UserAge is producing knowledge that can be used to inform research funders and politicians about the prerequisites needed to improve the efficiency of research about and with user involvement.

As to potential limitations, taking a critical stance, the fact that we ourselves are studying our previous and ongoing research deserves discussion. Carefully considering this, we designed the program to counteract threats to validity and trustworthiness. Bringing together researchers representing different disciplines, user representatives, and international experts early in the development of the program proposal and throughout the implementation of the program, there is a considerable critical mass. The Think Tank is functioning as an external resource, and the External Advisory Committee delivers input based on an instruction emphasizing their role as “critical friends.” Now midway into the program period, there are multiple examples of discursive standpoints that have emerged through an interchange of key topics and scientific controversies and agreements within and between the disciplines represented in UserAge. The importance of upholding a critical stance to the evolving program cannot be overestimated, and the mechanisms in place to counteract obvious limitations are promising to nurture knowledge generation on the empirical, capacity-building, and modeling levels.

Summing up, the UserAge program is an example of a major research endeavor with potential to inform research with and about user involvement in research on aging and health. Taken together and communicated in the international scientific community as well as in a wide range of public and policy arenas, the empirical results, capacity-building, and modeling efforts will have an impact not only on the present situation but also on the future.
